# Effect of positive pressure ventilation on lymphatic flow in pediatric patients

**DOI:** 10.1038/s41372-022-01563-7

**Published:** 2022-11-25

**Authors:** Sarah Khan, Christopher L. Smith, Erin M. Pinto, Dalal K. Taha, Kathleen A. Gibbs, Samuel A. Rosenblatt, Yoav Dori

**Affiliations:** 1 Department of Pediatrics, Einstein Medical Center, Philadelphia, PA 19141 USA; 2 Jill and Mark Fishman Center for Lymphatic Disorders, The Children’s Hospital of Philadelphia, Philadelphia, PA 19104 USA; 3 Division of Cardiology, The Children’s Hospital of Philadelphia and Department of Pediatrics Perelman School of Medicine at The University of Pennsylvania, Philadelphia, PA 19104 USA; 4 Division of Neonatology, The Children’s Hospital of Philadelphia and Department of Pediatrics, Perelman School of Medicine at The University of Pennsylvania, Philadelphia, PA 19104 USA; 5 Department of Anesthesiology and Critical Care Medicine, Children’s Hospital of Philadelphia and Department of Pediatrics Perelman School of Medicine at the University of Pennsylvania, Philadelphia, PA 19104 USA

**Keywords:** Interventional cardiology, Medical imaging, Adverse effects

## Introduction

Positive pressure ventilation (PPV) is used as an essential strategy for respiratory failure in the adult, pediatric and neonatal intensive care units but this is not without cost. The immediate effects of PPV on hemodynamics have been studied and one consequence is a significant increase in central venous pressure (CVP) [1]. In premature infants, long term PPV is also associated with the development of bronchopulmonary dysplasia (BPD) [2]. However, our understanding of the deleterious effects of PPV on other organ systems, including lymphatics, is more limited and isolated to animal studies. These studies have demonstrated increased lymphatic production and decreased lymphatic drainage due to PPV on lymphatic formation [3]. Other studies in animals have shown increased lymphatic flow and reduction in pulmonary edema with thoracic duct (TD) decompression [4].

While the animal data suggest that PPV may inhibit lymphatic flow there is little human data to support this physiology. This case series aims to describe the effect of PPV on lymphatic flow using lymphatic imaging techniques in four pediatric patients presenting for lymphatic studies due to lymphatic failure.

## Methods

This is a case series of four patients with severe BPD who underwent lymphangiography and TD outlet flow assessment. IRB approval was not required for this retrospective case series.

Dynamic contrast magnetic resonance lymphangiography (DCMRL) was performed on all patients as previously described [5]. Thoracic duct outflow (TDOF) was assessed by clearance of conventional or US contrast injection into the TD (Figure).

## Case series

All patients were admitted to a level 4 neonatal intensive care unit (NICU). All patients were born premature with a range of 24–33 weeks of gestational age. At the time of evaluation, they ranged between 0.3 and 15 months of chronologic age. Three patients received prolonged invasive PPV for severe BPD and one patient with prenatal hydrops required PPV due to a severe lymphatic conduction disorder. All patients had severe anasarca, with 2 having pleural effusions, and 3 having ascites.

**Fig. 1:**
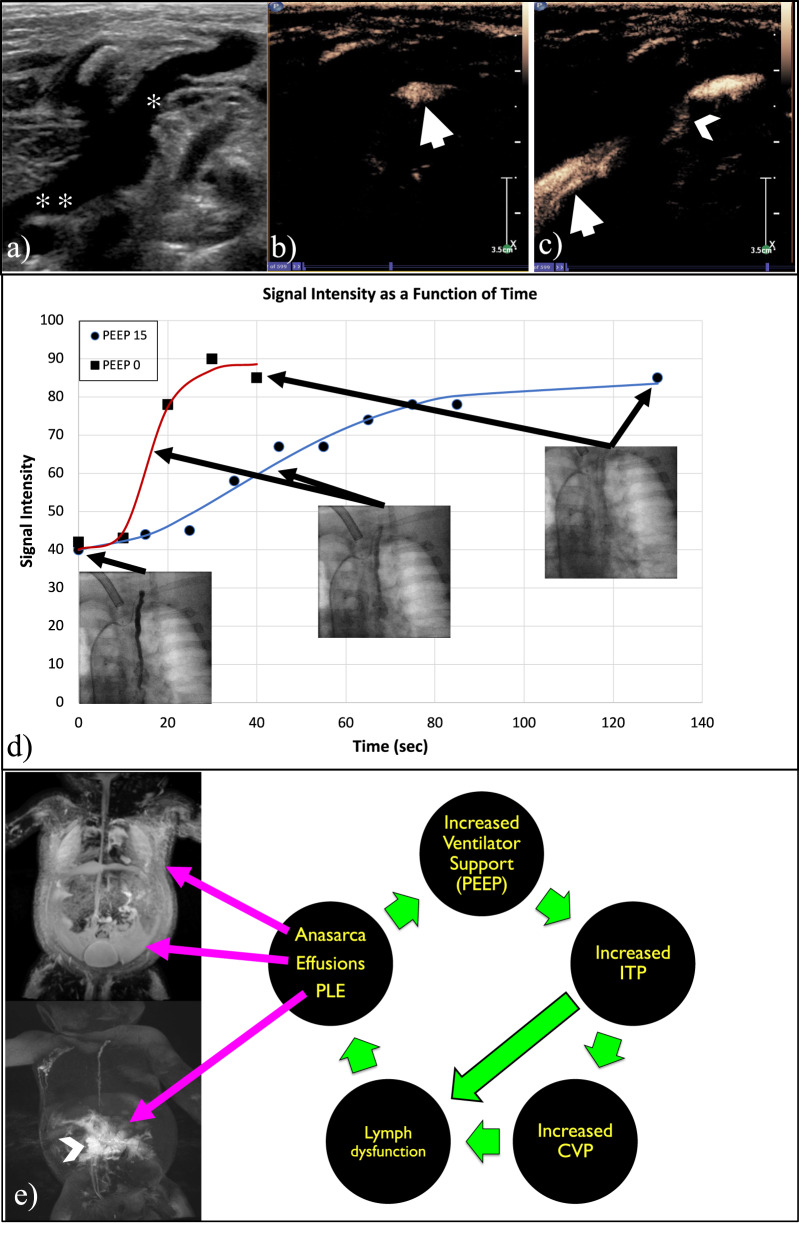
Comparison of TD emtying rates in high and low PEEP and the cycle of lymphatic failure with imaging of lymphatic failure consequences. **a** US of the jugular (✻) and innominate vein (✻ ✻). **b** Contrast US image showing filling of the TD outlet with contrast agent (arrow) without spilling into the innominate vein. **c** With PEEP off spilling of contrast (arrowhead) and filling of the innominate vein is seen. **d** Signal intensity as a function of time at the distal TD (inserts) (In this plot a value of 0 is black). **e** Depiction of the vicious cycle initiated by increased ventilatory support in patients with lymphatic dysfunction with insert showing effusions and edema on MIP T2 weighted coronal image (top) and MIP coronal of IH-DCMRL (bottom) demonstrating leak into the duodenum consistent with PLE (arrowhead).

Median PEEP was 17.5 (9–20) mmHg with median pressure support 23 (20–30) mmHg. At the baseline ventilator settings, the CVP median was 16.5 (7–18) mmHg and TD pressure in the 3 patients that it was measured was 17 (7–22) mmHg. With removal of PEEP, CVP measured 7 (4–13) mmHg and TD measured 8 (4–16) mmHg. At baseline ventilator settings, no or minimal TDOF was observed in all four patients. With removal of PEEP, TDOF improved and was brisk in all cases (Fig. 1a–d).

All patients underwent intranodal DCMRL (IN-DCMRL) and 2 patients also underwent intrahepatic DCMRL (IH-DCMRL) to evaluate for lymphatic abnormalities. All patients demonstrated poor antegrade lymphatic flow with evidence of pleural effusion, pulmonary edema, abdominal ascites, and dermal backflow through multiple cutaneous channels resulting in extensive body wall edema.

## Discussion

With the advent of novel lymphatic imaging and interventions, we were able to visualize the direct effects of removing positive pressure on the CVP and lymphatic flow through the TD in our patients. At baseline positive pressure settings, there was poor antegrade flow through the thoracic duct with pooling of contrast in the neck, congestion in the retroperitoneum and retrograde flow through multiple cutaneous channels. As we removed positive pressure, the TD pressure and CVP decreased. This resulted in improvement in thoracic duct flow with decreased contrast clearance time.

In physiologic respiratory cycle, negative intrathoracic pressure provides a favourable gradient for thoracic duct filling during inspiration that is then emptied during expiration. With the application of PPV, the CVP increases significantly increasing lymphatic production and reducing lymphatic drainage. This double hit on the lymphatic system can contribute to lymphatic failure which include symptoms such as anasarca, ascites, pleural effusions, and protein losing enteropathy. This creates a vicious cycle as shown in Fig. 1e.

In patients with known lymphatic conduction abnormalities, mild to moderate application of PEEP appears to worsen lymphatic flow and impede drainage as was observed in patient 4 with a lymphatic conduction disorder where a PEEP of 8 cm H_2_O was sufficient to impede lymphatic flow.

In conclusion, Novel lymphatic imaging techniques have allowed us to visualize the influence of positive pressure ventilation on the lymphatic system with a decrease in thoracic duct flow. It is important to recognize the full range of physiological derangements incurred and to be judicious with the amount of positive pressure delivered. In all patients requiring chronic PPV, but especially patients with underlying lymphatic conduction abnormalities, it is reasonable to use imaging to titrate positive pressure to the lowest possible to allow lymphatic clearance while maintaining adequate gas exchange and prevention of atelectasis.
